# An evolutionary optimization of a rhodopsin-based phototrophic metabolism in *Escherichia coli*

**DOI:** 10.1186/s12934-017-0725-6

**Published:** 2017-06-15

**Authors:** Hyun Aaron Kim, Hyun Ju Kim, Jihoon Park, Ah Reum Choi, Kyoo Heo, Haeyoung Jeong, Kwang-Hwan Jung, Yeong-Jae Seok, Pil Kim, Sang Jun Lee

**Affiliations:** 1 Hana Academy Seoul, Seoul, Republic of Korea; 2 Department of Systems Biotechnology, Chung-Ang University, Anseong, Gyeonggi Republic of Korea; 3 Department of Biotechnology, The Catholic University of Korea, Bucheon, Gyeonggi Republic of Korea; 4 Department of Life Sciences, Sogang University, Seoul, Republic of Korea; 5 Infectious Disease Research Center, Korea Research Institute of Bioscience and Biotechnology (KRIBB), Daejeon, Republic of Korea; 6 Department of Biological Sciences, Seoul National University, Seoul, Republic of Korea; 7 Department of Biological Sciences, Seoul National University, Seoul, Republic of Korea

**Keywords:** Adaptive laboratory evolution, Strain optimization, Chemotroph, Phototroph, Rhodopsin, Proton pumping

## Abstract

**Background:**

The expression of the *Gloeobacter* rhodopsin (GR) in a chemotrophic *Escherichia coli* enables the light-driven phototrophic energy generation. Adaptive laboratory evolution has been used for acquiring desired phenotype of microbial cells and for the elucidation of basic mechanism of molecular evolution. To develop an optimized strain for the artificially acquired phototrophic metabolism, an ancestral *E. coli* expressing GR was adaptively evolved in a chemostat reactor with constant illumination and limited glucose conditions. This study was emphasized at an unexpected genomic mutation contributed to the improvement of microbial performance.

**Results:**

During the chemostat culture, increase of cell size was observed, which were distinguished from that of the typical rod-shaped ancestral cells. A descendant ET5 strain was randomly isolated from the chemostat culture at 88-days. The phototrophic growth and the light-induced proton pumping of the ET5 strain were twofold and eightfold greater, respectively, than those of the ancestral *E. coli* strain. Single point mutation of C1082A at *dgcQ* gene (encoding diguanylate cyclase, also known as the *yedQ* gene) in the chromosome of ET5 strain was identified from whole genome sequencing analysis. An ancestral *E. coli* complemented with the same *dgcQ* mutation from the ET5 was repeated the subsequently enhancements of light-driven phototrophic growth and proton pumping. Intracellular c-di-GMP, the product of the diguanylate cyclase (*dgcQ*), of the descendant ET5 strain was suddenly increased while that of the ancestral strain was negligible.

**Conclusions:**

Newly acquired phototrophic metabolism of *E. coli* was further improved via adaptive laboratory evolution by the rise of a point mutation on a transmembrane cell signaling protein followed by increase of signal molecule that eventually led an increase proton pumping and phototrophic growth.

**Electronic supplementary material:**

The online version of this article (doi:10.1186/s12934-017-0725-6) contains supplementary material, which is available to authorized users.

## Background

Biological energy for life can be harvested from light, and converted into electrochemical proton gradient and/or ATP in phototrophic organisms such as plants and the cyanobacterium, *Gloeobacter violaceus* [1], using photosystems and rhodopsin-based mechanism [2], respectively. Rhodopsin is a proton-pumping transmembrane protein present in many cyanobacteria, and functions as a primitive photosystem [3]. Retinal, a prosthetic molecule present in rhodopsin, absorbs photons, triggers isomerization, and releases protons outside the cytoplasmic membrane [4]. Attention has been paid to the potential of the light-harvesting machinery because of its renewable use of solar energy in biological systems [5–7]. Phototrophic modules such as light-harvesting rhodopsin can be artificially transferred to chemotrophic cells to have additional light-driven energy metabolism. For example, light illumination on a proteorhodopsin-integrated membrane in *E. coli* resulted in the generation of a proton motive force that can promote flagellar motility [8]. The coupling of the light-driven proton-pumping *G. violaceus* rhodopsin (GR) and *E. coli* ATP synthase in the same membrane could generate ATP production [9].

Adaptive laboratory evolution (ALE) has been harnessed for the elucidation of basic mechanism of molecular evolution and genome dynamics, and the direction of wanted phenotypes of microbial cells [10]. In application aspects, evolved mutations would allow the optimization of microbial fitness, and they could be transferred to other backgrounds hosts for the acquiring of new cellular functions, which are named evolutionary engineering and reverse metabolic engineering, respectively [11, 12]. Microbial mutations could increase biotechnological productivity and yield [13–15]. Adaptive laboratory evolutions could allow microbial strains to obtain industrially beneficial characteristics such as tolerance to higher concentrations of substrate or product, stress tolerance against toxic chemicals, etc. [16–18]. Chemostat cultures have been preferred to simple serial batch transfer in evolutionary experiments, because environmental factors such as nutrients, pH, oxygenation, and growth rate could be maintained [19].

In this study, a phototrophic module (i.e., GR: *Gloeobacter* rhodopsin) was introduced into a chemotrophic *E. coli* host, and evolution of the phototrophic metabolism was induced under illumination condition by chemostat. The improvement of light-driven proton pumping and phototrophic growth were observed in the descendant strain, in which the corresponding genomic mutation was characterized by genome sequencing analysis and confirmed by genomic complementation. The physiological characteristics of the evolved cells and the evolutionary direction of new phototrophic metabolism were also discussed.

## Methods

### Strain, medium, and adaptive evolution

An *E. coli* W3110 (laboratory stock at the Catholic University of Korea) harboring pKJ606-GR plasmid [20] was used as the ancestral strain for adaptive evolution. Chemostat culture of the *E. coli* ancestral strain was performed using modified M9 minimal medium under illumination condition. The minimal medium composition was as follows: 1 g/L glucose, 0.8 g/L NH_4_Cl, 0.5 g/L NaCl, 7.5 g/L Na_2_HPO_4_·2H_2_O, 3 g/L KH_4_PO_4_, 0.2 g/L MgSO_4_·7H_2_O, 0.1 g/L CaCl_2_, 1 mg/L thiamine·HCl supplemented with 5 μM all-*trans*-retinal (dissolved in ethanol, Cat. number R-2500, Sigma-Aldrich Co. St. Louis, MO, USA), 50 μg/mL ampicillin, and 0.1 mM IPTG.

A single colony of the ancestral *E. coli* was inoculated in 3 mL of the minimal medium in a 15 mL tube, and incubated at 37 °C and 200 rpm for 16 h. Then, 1 mL of the culture broth was transferred to a 250 mL mini-chemostat fermenter jar (Hanil Inc., Gimpo, Korea) containing 100 mL of medium and equipped with LED light bulbs (four 1-W bulbs at 1 cm distances). The mini-fermenter was operated at 37 °C and 200 rpm with aeration (100 mL/min) and constant illumination. A 20 L reservoir was replenished with fresh feeding medium of the same composition as the initial medium whenever depleted. The reservoir jar was wrapped with aluminum foil to reduce inactivation of the light-sensitive retinal component. Inlet and outlet tubings were controlled by peristaltic pumps at 10 mL/h (corresponding to a dilution rate of 0.1 h^−1^). Samples (1 mL) were collected through the outlet tubing to measure optical density at 600 nm (OD_600_) [21].

### Electron microscopy

Samples from the outlet tubing during the chemostat operation were observed daily under a binocular microscope (BX41, Olympus, Tokyo, Japan) at 1000× magnification. The chemostat culture populations and an evolved descendant *E. coli* were observed by scanning electron microscopy (S-4800, Hitachi, Tokyo, Japan) in a chemical analytical facility at the Catholic University of Korea.

The chemostat culture broth at 88-days was spread on the agar (1.5%) containing minimal medium. After incubation for 48 h at 37 °C with illumination (13-W bulb at 10 cm distance), a single colony was randomly selected (named ET5) and stored for further analysis.

### Phototrophic growth measurement

To estimate the phototrophic growth of the ancestral and the evolved *E. coli* strains, cells were batch-cultured in a 250 mL Erlenmeyer flask containing 50 mL of fresh medium at 37 °C and 200 rpm for 24 h in a shaking incubator equipped with two 13-W light bulbs at a distance of 20 cm. The same medium composition of the chemostat was used, except glucose concentration (2 g/L) for higher biomass. Cell growth was observed by measuring OD_600_. Chemotrophic growth was measured under light-protected conditions by wrapping the flask with aluminum foil. Phototrophic growth was calculated by subtracting cell growth (OD_600_) in dark condition from cell growth (OD_600_) under illumination condition. The growth measurement was repeated at least three times.

### Proton pumping measurement

To measure the amount of proton pumping by GR, the cells in log phase were harvested by moderate centrifugation (3600 rpm for 10 min) at room temperature. The cell pellet was gently washed twice with unbuffered saline solution (10 mM NaCl, 10 mM MgSO_4_·7H_2_O, and 0.1 mM CaCl_2_) [23]. Samples were placed in a spectrophotometric cuvette (path length 1 cm) and illuminated at an intensity of 100 W/m^2^. Illumination was given using the short-wave cutoff filter (>440 nm, Sigma Koki SCF-50S-44Y, Japan) in combination with a focusing convex lens and heat-protecting CuSO_4_ filter. Proton pumping was monitored for 1 min by measuring pH values using the F-51 pH meter (Horiba Ltd., Kyoto, Japan) [22]. The measurement was repeated at least three times, and the difference in proton concentrations was determined and fitted with pH = −log_10_[H^+^] with calibration by acid addition [22].

### Genome analysis

Genomic and plasmid DNAs of the ancestral and the evolved strains were purified using a Wizard genomic DNA purification kit (Promega, Madison, WI, USA) and a Plasmid miniprep kit (Qiagen, Hilden, Germany), respectively. The purified genomic and plasmid DNA samples were mixed in a ratio of 100:1 by mass, and were subjected to sequencing library construction with an average insert size of 600 bp using Illumina TruSeq Nano DNA kit (San Diego, CA, USA). A 300-cycle paired-end sequencing was performed in a sequencing facility at KRIBB (Daejeon, Korea) using an Illumina MiSeq platform (San Diego, CA, USA). Read preprocessing (quality trim limit 0.01, one ambiguous nucleotide allowed per read, and minimum read length of 100 bp), reference mapping, and fixed ploidy variant detection (including indels and structural variants detection) were all conducted using the CLC Genomics Workbench 8.0. The complete genome sequence of *E. coli* W3110 (RefSeq NC_007779.1) and the sequence of plasmid pKJ606-GR [20] were used as the references.

### Genomic complementation of the single point mutation

*Escherichia coli* JW1912 strain carrying the kanamycin-resistance gene (KmR) in the place of the open reading frame of *amyA* encoding α-amylase, which is located 20 kb from the point mutation in the chromosome, was obtained from the Keio collection [23]. The P1 lysate of JW1912 cells was used to transduce Δ*amyA*::KmR into the ET5 strain to make KmR-tagged *dgcQ* ^C1082A^ strain (HK769), which was verified by DNA sequencing. Subsequently, the P1 lysate of HK769 was used to transduce the point mutation of *dgcQ* ^C1082A^ into the ancestral strain to generate isogenic HK775 strain.

### Determination of intracellular c-di-GMP concentration

Intracellular c-di-GMP concentration was determined according to previous publication [24]. Parental type (W3110/GR) and the evolved type (ET5/GR) cells were grown in the M9-glucose minimal medium supplemented with 5 μM all-trans-retinal (dissolved in ethanol), and 50 μg/mL ampicillin under dark or illuminated conditions. When OD reached about 0.5, the culture broths (50 mL) were subjected to centrifugation (at 6000×*g* and 4 °C, for 10 min). The cells were rapidly frozen by liquid nitrogen after discarding the supernatant and stored at a deep freezer (−80 °C) until extraction. The frozen sample was thawed and mixed with an pre-chilled extraction buffer (methanol:acetonitrile:0.1 N formic acid solution = 40:40:20; 500 μL) and an internal standard (3′,5′-cyclic xanthosine monophosphate; 5 μL). The tube containing the mixture was tightly sealed, then subjected into freezing and thawing to disrupt cells using a liquid nitrogen and a heating block (30 °C) for three times. The tube was incubated in an ice bath for 30 min before the centrifugation (at 10,000×*g*, 4 °C, for 10 min). The supernatant (400 μL) was transferred into a 1.5 mL-tube and concentrated using a vacuum centrifuge (55 °C), followed by triple distilled water (TDW; 100 μL) addition to resuspend. The suspension sample was centrifuged at 10,000×*g*, 4 °C, for 30 min to remove debris, and the supernatant was stored at −80 °C. The c-di-GMP concentration in the supernatant was determined by a LC-MS/MS (Accela 1250 UPLC system, Thermo Fisher Scientific, Waltham, MA, USA) equipped with a C18 column (Hypersil Gold column, 2.1 × 100 mm, 1.8 μm, Thermo Fisher Scientific) and a mass spectrometer (TSQ Quantum Access MAX Quadrupole MS, Thermo Fisher Scientific) at a chemical analysis facility (Seoul National University). Standard chemicals of c-di-GMP and 3′,5′-cyclic xanthosine monophosphate (cXMP) were purchased from Sigma-Aldrich Co.

## Results

### Adaptive evolution of GR-expressing *E. coli*

**Fig. 1:**
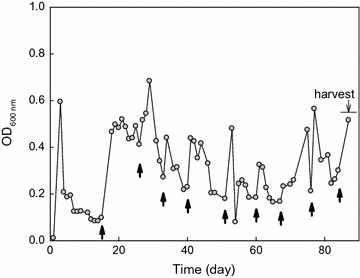
Long-term chemostat adaptive laboratory evolution of GR-expressing *E. coli* with illumination. *Upward arrows* indicate when the reservoir was exchanged with fresh medium

Chemotrophic *E. coli* W3110 expressing GR exhibits phototrophic ATP generation. For adaptive evolution, the ancestral strain (*E. coli* W3110 carrying pKJ606-GR) was grown in a chemostat reactor containing minimal medium with limited d-glucose (1 g/L) as the sole carbon source under constant illumination condition. In the constantly illuminated chemostat culture, optical density (OD_600_) of the culture broth was increased whenever the reservoir was replenished with fresh medium then gradually decreased (Fig. 1; upward arrows for reservoir replenishment). The OD_600_ was maintained in between 0.1 and 0.7 during the chemostat operation. The residual glucose in the outlet tubing was not detected. Two other chemostat cultures of the ancestral strain were also operated under the same medium conditions, but with different illumination conditions (lights were switched on and off every 15 min in one culture, and every 12 h in the other culture); these cultures became washed out after 10 days.

**Fig. 2:**
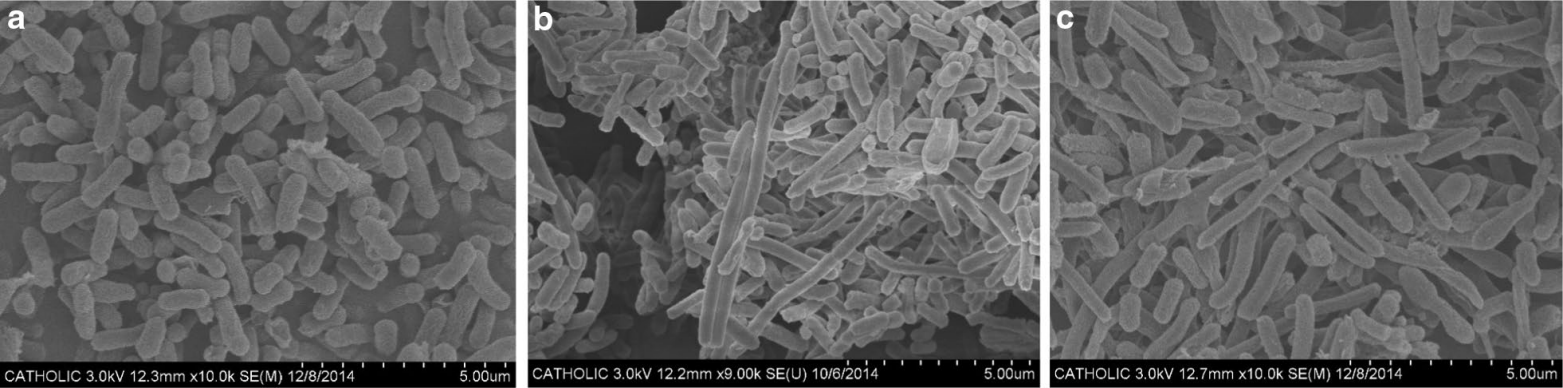
Scanning electron microscopic images of the population of chemostat-cultured *E. coli* cells expressing GR under illumination condition. **a** Ancestral *E. coli* expressing GR (×10,000 magnification). **b** Population of the evolved *E. coli* expressing GR after 30 days of the chemostat culture (×9000 magnification). **c** Population of the evolved *E. coli* expressing GR after 88 days of the chemostat culture (×10,000 magnification)

Using optical microscopy, elongated cells were observed after 15 days. The population of slightly elongated cells was gradually increased during the chemostat operation, and most of the microscopic observed cells were elongated at 88-days. Morphological characteristics of cell populations after 30-days (corresponding to 100 generations) and 88-days (corresponding to 300 generations) were further examined using scanning electron microscopy (Fig. 2). The ancestral cell was typically rod-shaped with a long axis in the range of 1.5–2.2 µm (Fig. 2a). After 30-days, atypical longer cells were observed (Fig. 2b) along with the wild-type sized cells. The longest cell observed in the culture sample had a long axis of 9.3 µm (similar with short axis of 0.5 µm). After 88-days (Fig. 2c), the population of the cells with the atypical morphology increased when compared to the population of cells after 100 generations. Among the 88-days evolved *E. coli* population, a single colony was randomly selected and named *E. coli* ET5 (represents 5-μm-long evolved type).

### Phototrophic growth of ET5 strain

The ancestral strain and evolved ET5 cells were grown in the minimal medium with and without illumination (Table 1). After cultivation for 24 h, residual glucose in culture medium was not detected. The growth of *E. coli* W3110 was not affected by the presence or absence of light, due to the lack of phototrophic machinery in the cell. The cell growth of ancestral strain was slightly increased to 1.75 of OD_600_ by light, compared to 1.52 of OD_600_ in the absence of light. The estimated phototrophic growth of the ancestral strain was 0.23 of OD_600_. In case of the descendant ET5, the phototrophic growth was calculated as 0.51 of OD_600_, which was 2.2-fold greater than that of the ancestral strain. This data implied that the ET5 cells have been evolved from the ancestral strain during the chemostat culture to grow efficiently under illumination condition.

**Table 1:** Cell growths of rhodopsin-expressing *E. coli* strains with and without illumination

Strain	Description	Cell growth (OD_600_)^a^		Phototrophic growth Δ[B − A] (fold)
		In dark (A)	In light (B)	
W3110	Negative control	1.78 ± 0.05	1.78 ± 0.04	0^b^
W3110/pKJ606-GR	Ancestral strain	1.52 ± 0.05	1.75 ± 0.08	0.23 (1.0)
ET5/pKJ606-GR	Evolved strain	1.52 ± 0.00	2.03 ± 0.08	0.51 (2.2)

^a^Data represent the mean of optical densities ± SD at 600 nm from at least three repeats

^b^Wild-type *E. coli* W3110 did not show any difference between growth in dark (A) and growth in light (B)

### Morphology and genomic variation of ET5 strain

**Fig. 3:**
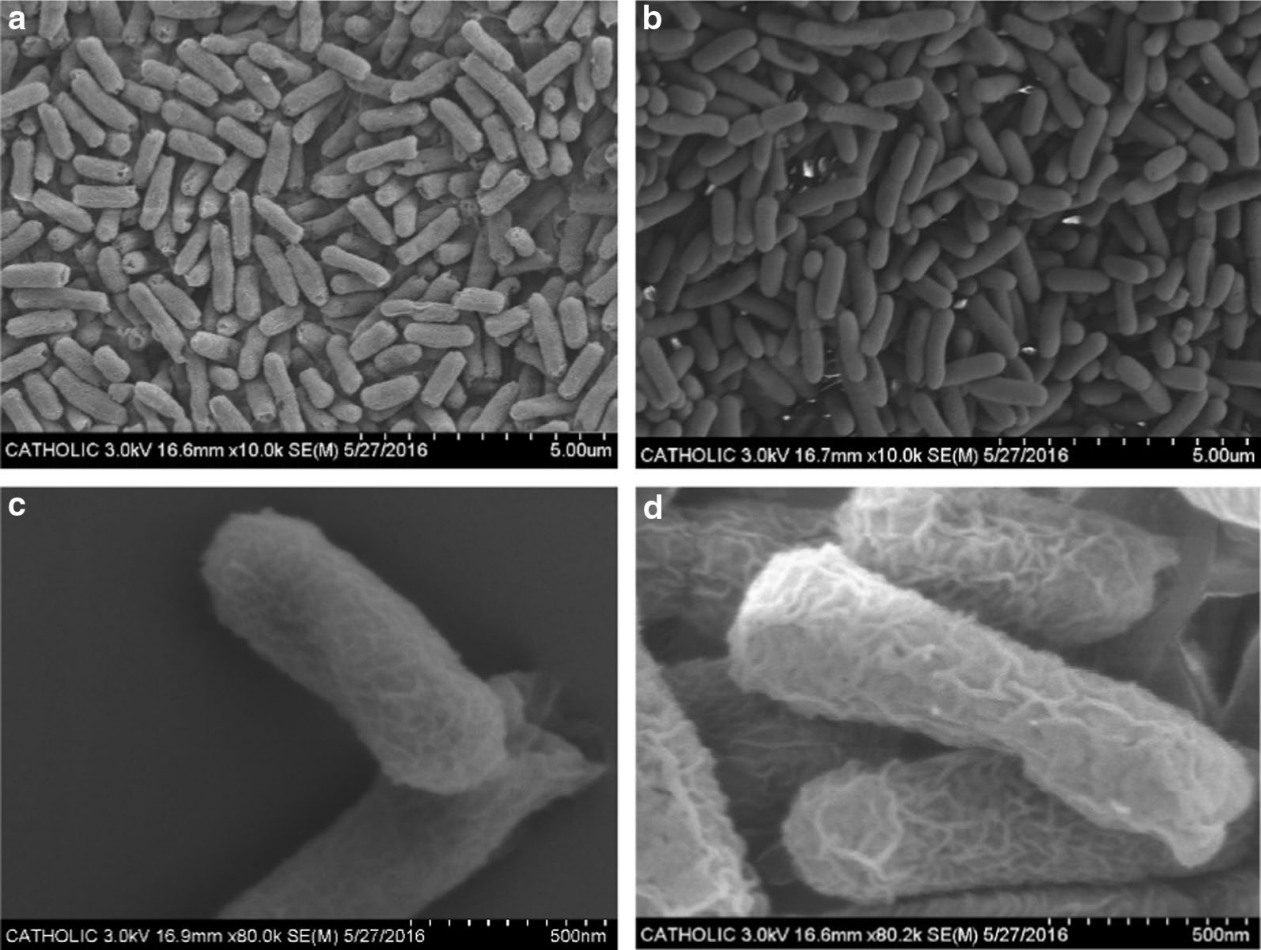
Scanning electron microscope images of the batch-cultured *E. coli* ET5. **a** Ancestral strain ×10,000. **b** ET5 ×10,000. **c** Ancestral strain ×80,000. **d** ET5 ×80,000

As the cell population in the chemostat showed an atypical elongated shape during the adaptive laboratory evolution, the isolated ET5 strain was observed in detail after batch culture (Fig. 3). No elongated cells of ET5 were found in the 10,000-fold magnified scanning electron microscopic images (Fig. 3a). The 80,000-fold magnified images (Fig. 3c, d) revealed that ET5 had a slightly more wrinkled surface structure compared to the surface of the ancestral strain (Additional file 1: Figure S1).

The whole genome and harboring plasmid of the ancestral strain and ET5 were sequenced with reference to the genome of *E. coli* W3110 (NC_007779.1). The ancestral strain *E. coli* W3110 (laboratory stock at the Catholic University of Korea) has several differences when compared to the published reference sequence. Variations including 16 single nucleotide variants (SNV), 4 short indels, and a 15.2 kb-deletion of e14 prophage region (probably caused by the crossover between *icd* and *icdC*), co-existed throughout all the samples, and thus, they were excluded from the candidate genes that can elucidate the newly acquired characteristics in ET5 (Additional file 1: Table S1, Figure S2). The ET5 showed no mutations on the plasmid. Compared to the ancestral strain, the strain ET5 only have a single nucleotide point mutation (C1082A) on the structural gene of *dgcQ*, which corresponds to the substitution of single amino acid (A361E) in a diguanylate cylase encoded by *dgcQ* gene (Table 2).

**Table 2:** Genome sequencing and mapping summary

Strain	DNA	Mutation	Average coverage	Reads (trimmed) ×10^6^	Bases (trimmed) ×10^6^	Avg. length (trimmed)	Read mapping rate (%)
Ancestral strain	Chromosome	–	479	13.2	2754	208	99.41
	Plasmid	–	58,504				
ET5	Chromosome	*dgcQ* ^C1081A^	468	14.2	2980	210	99.50
	Plasmid	–	90,534				

Trim rates based on read numbers were 87.48 and 88.79%, respectively

### Comparison of light-driven proton pumpings

**Fig. 4:**
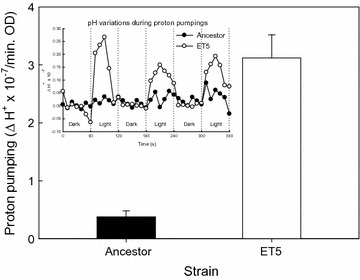
Light-driven proton pumping in the *E. coli* strains expressing GR. The ancestral *E. coli* cells expressing GR and ET5 evolved from the ancestral cells were suspended in an unbuffered saline solution, and proton pumping was estimated by measuring the pH decrease after 60-s illumination. *Bar grap*h represents the proton pumping as mean ± SD from at least three measurements, and the *line-scattered graph* indicates the pH traces during the measurement (*Closed bar/circle* ancestral strain, *Open bar/circle* ET5)

Based on the greater phototrophic growth of the evolved ET5 strain than the ancestral strain, it was hypothesized that the evolved ET5 strain might have a more efficient phototrophic energy-producing metabolism. To verify this hypothesis, the light-induced proton pumping efficiency was determined by measuring the pH decrease in an unbuffered saline solution (Fig. 4). The proton pumping of the ancestral strain was 0.38 (extracellular ΔH^+^ × 10^−7^/min OD), whereas that of ET5 was 3.12 (extracellular ΔH^+^ × 10^−7^/min OD). The light-induced proton pumping efficiency of the ET5 was eightfold greater than that of the ancestral strain. This result showed that ET5 has been evolved to have a highly efficient proton pumping.

To verify the *dgcQ* ^C1082A^ point mutation was responsible to the increase of the light-driven proton pumping, the *dgcQ* ^C1082A^ mutation was introduced into the chromosome of the an ancestral strain (W3110 *amyA*::KmR; HK774 strain) and the phototrophic characteristics of the complemented strain were measured (Table 3). The phototrophic growth of the ancestral *E. coli amyA*::KmR *dgcQ* ^C1082A^ (HK775 strain) was greater (0.51 OD_600 nm_) than that of the ancestral *E. coli amyA*::KmR *dgcQ* ^WT^ (HK774 strain; 0.10 OD_600 nm_). The proton pumping of complemented strain also agreed with the increase in the phototrophic growth by the point mutation, those were 0.48 for HK774 and 0.90 for HK775 (extracellular ΔH^+^ × 10^−7^/min OD), respectively. The proton pumping efficiency of HK775 (*dgcQ* ^C1082A^) was enhanced twice when compared to that of HK774 (*dgcQ* ^WT^). This complementation experiment indicates that the point mutation of *dgcQ* gene was mainly responsible for the efficient light-driven proton pumping in the evolved ET5 strain. No distinctive morphology variation was found between the HK775 and the ET5 in the light microscopic observation.

**Table 3:** Effect of evolution-induced *dgcQ* ^C1081A^ mutation on phototrophic growth and proton pumping

Strain	Genotype	Growth in dark (A)	Growth in light (B)	Phototrophic growth Δ[B − A]^a^	Proton pumping (ΔH^+^ × 10^−7^/min OD)
HK774	Ancestral *E. coli amyA*::KmR, *dgcQ* ^WT^	0.98 ± 0.02	1.08 ± 0.04	0.10	0.475 ± 0.020
HK775	Ancestral *E. coli amyA*::KmR, *dgcQ* ^C1082A^	1.13 ± 0.04	1.64 ± 0.03	0.51	0.903 ± 0.107

^a^Phototrophic growth was estimated from the difference of the growth in light conditions (B) and the growth in dark conditions (A). Data of the growths represent the mean of optical densities ± SD at 600 nm from at least three biological repeats

### Comparison of c-di-GMP levels

Since it was verified that the *dgcQ* ^C1082A^ was the reason of the increase of phototrophic abilities (Tables 1, 3; Fig. 4), the variation of the responding metabolite of the *dgcQ* gene, c-di-GMP, was measured using LC–MS/MS (Table 4). The c-di-GMP of the ancestral strain grown under dark conditions was less than 0.03 μM/OD, whereas that of the evolved strain was 6.46 μM/OD. Same pattern was observed from the cells grown under light conditions, that was, c-di-GMP of ancestral strain was less than 0.02 μM/OD and that in the evolved strain was 7.16 μM/OD. Therefore, the mutation *dgcQ* ^C1082A^ led the abrupt increase of the responding signal molecule, c-di-GMP.

**Table 4:** Effect of evolution-induced *dgcQ* ^C1082A^ mutation on intracellular c-di-GMP concentrations

Strain	Genotype	c-di-GMP (μM/OD)	
		Dark conditions	Light conditions
Ancestral strain	*E. coli* W3110 *dgcQ* ^WT^/pKJ606-GR	<0.03	<0.02
Evolved strain	*E. coli* W3110 *dgcQ* ^C1082A^/pKJ606-GR	6.46 ± 3.43	7.16 ± 2.40

## Discussion

The *Gloeobacter* rhodopsin-introduced chemotrophic *E. coli* was adaptively evolved to be more susceptible to phototrophic energy metabolism. A single point mutation on chromosome (Table 2) enhanced light-induced proton pumping (Fig. 4), and the increase of phototrophic-generated energy in the descendant *E. coli* resulted in the enhancement of growth in the light (Table 1). Therefore, due to evolution, the ancestral *E. coli* was able to enhance the ability of the newly acquired phototrophic metabolism in its descendants.

As there was no mutation on the plasmid-origin genes, GR protein itself was identical between the ancestral strain and the descendants (Table 2). The ET5 harbored mutated *dgcQ* gene (also known as the *yedQ* gene) that encodes a putative diguanylate cyclase which is involved in the regulation of bacterial cell surface-associated traits including biofilm formation [25] and swimming motility [26] via c-di-GMP, a second messenger in signal transduction [27]. In addition, a recent study predicted a periplasmic cyclase/histidine kinase-associated sensory domain in DgcQ [28]. The surface variations derived from the c-di-GMP fluctuation in ET5 would have enhanced the phototrophic proton pumping efficiency of rhodopsin by eightfold, as compared to the ancestral strain (Fig. 4). The authors have presumed the mutation on DgcQ might have affected the intracellular level of c-di-GMP, which was indeed confirmed the sudden increase in the evolved strain (Table 4). The increase of c-di-GMP would have signaled on various gene expressions leading modulations of cell surface physiology, or might have interacted directly with GR in the cellular membrane, resulting in enhanced proton pumping in the evolved ET5 strain.

The *dgcQ* ^C1082A^-induced proton pumping increase in the W3110 host was eightfold (Fig. 4) whereas that in the *amyA* mutant host (HK774) was only twofold (Table 3). It is not clear why the *amyA* mutant host only showed marginal proton pumping enhancement. It could not be excluded the possibility that the absence of *amyA* encoding α-amylase and therefrom metabolic variation in the *amyA* mutant host background might have contradicted the *dgcQ* ^C1082A^-induced proton pumping increase in unknown mechanism.

An elongated cell that was observed during the chemostat culture (Fig. 2) must have had evolutionary merit over a non-elongated cell because the widened surface area would have provided more phototrophic energy generation. It was not clear why elongated cells during the chemostat culture were not distinctively observed in the batch culture of ET5 (Fig. 3; Additional file 1: Figure S1). Elongated bacterial shape has been observed in stressed conditions such as oxidative stress, nutrient limitation, DNA damages, antibiotics exposure, or extensive recombinant protein expression [29–31] altering DNA replication and cell division. The plausible reason for this might be that the *E. coli* might have recognized stress and exhibited elongated cell morphology only within a narrow range of stress conditions, i.e., when the growth rate during the chemostat was D = 0.1 h^−1^, corresponding to early stationary phase, while the SEM observation and flow cytometry analysis were performed using mid log phase cells in batch cultures. Another explanation for this might be that the elongated cells during the chemostat culture were not selected in the isolation process by chance.

It is noteworthy that the ancestral strain from the home stock, which we assumed it would be similar to the reference wild-type W3110 strain at the beginning of this study, already carried several mutations (Additional file 1: Table S1, Figure S2). These mutations must have accumulated during the laboratory passages and must have been beneficial for its growth in the laboratory conditions, which is representative of bacterial genome plasticity [32, 33].

## Conclusions

The artificially introduced phototropic energy metabolism of *E. coli* has been evolved by the chemostat, indicating the efficiency of synthetic biological parts could be further optimized to some extent via adaptive laboratory evolution. We figured out the single point mutation of a membrane protein involved in cell signal transduction was responsible for the enhanced light-driven cellular performance. Our study is at the beginning stage in the development of artificial phototropism in chemotrophic cells, and further investigation is required to utilize infinite light energy efficiently in synthetic biological systems.

## Additional file

**Additional file 1: Figure S1.** Distribution of cell size in the batch-cultured ET5 strain compared with the ancestral strain by flow cytometry. **Table S1.** List of all variations in the W3110 ancestral strain identified using NC_007779.1 as a reference. **Figure S2.** A 15.2-kb deletion identified as a zero-coverage region after mapping analysis. It might have been caused by a crossover between *icd* (Y75_p1106, 1251 bp) and *icdC* (pseudo; Y75_p1130, 153 bp). Sequence alignment showed that *icdC* is the C-term part of *icd* (93% nucleotide identities). The image was captured from the output screen of the CLC genomics workbench.

## References

[1] Belkin S, Mehlhorn RJ, Packer L (1987). Proton gradients in intact cyanobacteria. Plant Physiol.

[2] Imasheva ES, Balashov SP, Choi AR, Jung KH, Lanyi JK (2009). Reconstitution of *Gloeobacter violaceus* rhodopsin with a light-harvesting carotenoid antenna. Biochemistry.

[3] Nakamura Y, Kaneko T, Sato S, Mimuro M, Miyashita H, Tsuchiya T, Sasamoto S, Watanabe A, Kawashima K, Kishida Y (2003). Complete genome structure of *Gloeobacter violaceus* PCC 7421, a cyanobacterium that lacks thylakoids. DNA Res.

[4] Pinhassi J, DeLong EF, Beja O, Gonzalez JM, Pedros-Alio C (2016). Marine bacterial and archaeal ion-pumping rhodopsins: genetic diversity, physiology, and ecology. Microbiol Mol Biol Rev.

[5] Claassens NJ, Volpers M, dos Santos VA, van der Oost J, de Vos WM (2013). Potential of proton-pumping rhodopsins: engineering photosystems into microorganisms. Trends Biotechnol.

[6] Kim JY, Jo BH, Jo Y, Cha HJ (2012). Improved production of biohydrogen in light-powered *Escherichia coli* by co-expression of proteorhodopsin and heterologous hydrogenase. Microb Cell Fact.

[7] Lee HJ, Park J, Lee JY, Kim P (2016). A strategy to increase microbial hydrogen production, facilitating intracellular energy reserves. J Microbiol Biotechnol.

[8] Walter JM, Greenfield D, Bustamante C, Liphardt J (2007). Light-powering *Escherichia coli* with proteorhodopsin. Proc Natl Acad Sci USA.

[9] Choi AR, Shi L, Brown LS, Jung KH (2014). Cyanobacterial light-driven proton pump, *Gloeobacter* rhodopsin: complementarity between rhodopsin-based energy production and photosynthesis. PLoS ONE.

[10] Dragosits M, Mattanovich D (2013). Adaptive laboratory evolution—principles and applications for biotechnology. Microb Cell Fact.

[11] Wang L, Spira B, Zhou Z, Feng L, Maharjan RP, Li X, Li F, McKenzie C, Reeves PR, Ferenci T (2010). Divergence involving global regulatory gene mutations in an *Escherichia coli* population evolving under phosphate limitation. Genome Biol Evol.

[12] Pal C, Papp B, Posfai G (2014). The dawn of evolutionary genome engineering. Nat Rev Genet.

[13] Mormann S, Lomker A, Ruckert C, Gaigalat L, Tauch A, Puhler A, Kalinowski J (2006). Random mutagenesis in *Corynebacterium glutamicum* ATCC 13032 using an IS6100-based transposon vector identified the last unknown gene in the histidine biosynthesis pathway. BMC Genomics.

[14] Zanfardino A, Restaino OF, Notomista E, Cimini D, Schiraldi C, De Rosa M, De Felice M, Varcamonti M (2010). Isolation of an *Escherichia coli* K4 *kfoC* mutant over-producing capsular chondroitin. Microb Cell Fact.

[15] Zhou X, Zhu H, Liu L, Lin J, Tang K (2010). A review: recent advances and future prospects of taxol-producing endophytic fungi. Appl Microbiol Biotechnol.

[16] Lee JY, Lee HJ, Seo J, Kim ES, Lee HS, Kim P (2014). Artificial oxidative stress-tolerant *Corynebacterium glutamicum*. Amb Express.

[17] Lee JY, Seo J, Kim ES, Lee HS, Kim P (2013). Adaptive evolution of *Corynebacterium glutamicum* resistant to oxidative stress and its global gene expression profiling. Biotechnol Lett.

[18] Kwon YD, Kim S, Lee SY, Kim P (2011). Long-term continuous adaptation of *Escherichia coli* to high succinate stress and transcriptome analysis of the tolerant strain. J Biosci Bioeng.

[19] Barrick JE, Lenski RE (2013). Genome dynamics during experimental evolution. Nat Rev Genet.

[20] Lee KA, Jung KH (2011). ATP regeneration system using *E. coli* ATP synthase and *Gloeobacter* rhodopsin and its stability. J Nanosci Nanotechnol.

[21] Jeong H, Lee SJ, Kim P (2016). Procedure for adaptive laboratory evolution of microorganisms using a chemostat. J Vis Exp.

[22] Wang WW, Sineshchekov OA, Spudich EN, Spudich JL (2003). Spectroscopic and photochemical characterization of a deep ocean proteorhodopsin. J Biol Chem.

[23] Baba T, Ara T, Hasegawa M, Takai Y, Okumura Y, Baba M, Datsenko KA, Tomita M, Wanner BL, Mori H (2006). Construction of *Escherichia coli* K-12 in-frame, single-gene knockout mutants: the Keio collection. Mol Syst Biol.

[24] Irie Y, Parsek MR (2014). LC/MS/MS-based quantitative assay for the secondary messenger molecule, c-di-GMP. Methods Mol Biol.

[25] Sanchez-Torres V, Hu H, Wood TK (2011). GGDEF proteins YeaI, YedQ, and YfiN reduce early biofilm formation and swimming motility in *Escherichia coli*. Appl Microbiol Biotechnol.

[26] Pesavento C, Becker G, Sommerfeldt N, Possling A, Tschowri N, Mehlis A, Hengge R (2008). Inverse regulatory coordination of motility and curli-mediated adhesion in *Escherichia coli*. Genes Dev.

[27] Boehm A, Kaiser M, Li H, Spangler C, Kasper CA, Ackermann M, Kaever V, Sourjik V, Roth V, Jenal U (2010). Second messenger-mediated adjustment of bacterial swimming velocity. Cell.

[28] Hengge R, Galperin MY, Ghigo JM, Gomelsky M, Green J, Hughes KT, Jenal U, Landini P (2015). Systematic nomenclature for GGDEF and EAL domain-containing cyclic di-GMP turnover proteins of *Escherichia coli*. J Bacteriol.

[29] Costa SB, Campos AC, Pereira AC, de Mattos-Guaraldi AL, Junior RH, Rosa AC, Asad LM (2012). The role of DNA base excision repair in filamentation in *Escherichia coli* K-12 adhered to epithelial HEp-2 cells. Antonie Van Leeuwenhoek.

[30] Justice SS, Hunstad DA, Cegelski L, Hultgren SJ (2008). Morphological plasticity as a bacterial survival strategy. Nat Rev Microbiol.

[31] Miller C, Thomsen LE, Gaggero C, Mosseri R, Ingmer H, Cohen SN (2004). SOS response induction by beta-lactams and bacterial defense against antibiotic lethality. Science.

[32] Escudero JA, Loot C, Parissi V, Nivina A, Bouchier C, Mazel D (2016). Unmasking the ancestral activity of integron integrases reveals a smooth evolutionary transition during functional innovation. Nat Commun.

[33] Vijayendran C, Polen T, Wendisch VF, Friehs K, Niehaus K, Flaschel E (2007). The plasticity of global proteome and genome expression analyzed in closely related W3110 and MG1655 strains of a well-studied model organism, *Escherichia coli*-K12. J Biotechnol.

