# Supplementary material for: An evolutionary optimization of a rhodopsin-based phototrophic metabolism in Escherichia coli
Source: Microb Cell Fact. 2017 Jun 15;16:111. doi: 10.1186/s12934-017-0725-6 (PMC5472908; doi:10.1186/s12934-017-0725-6)
Supplement: Supplementary file 1 — Additional file 1: Figure S1. Distribution of cell size in the batch-cultured ET5 strain compared with the ancestral strain by flow cytometry. Table S1. List of all variations in the W3110 ancestral strain identified using NC_007779.1 as a reference. Figure S2. A 15.2-kb deletion identified as a zero-coverage region after mapping analysis. It might have been caused by a crossover between icd (Y75_p1106, 1251 bp) and icdC (pseudo; Y75_p1130, 153 bp). Sequence alignment showed that icdC is the C-term part of icd (93% nucleotide identities). The image was captured from the output screen of the CLC genomics workbench. [file 12934_2017_725_MOESM1_ESM.docx]

**Figure S1**.


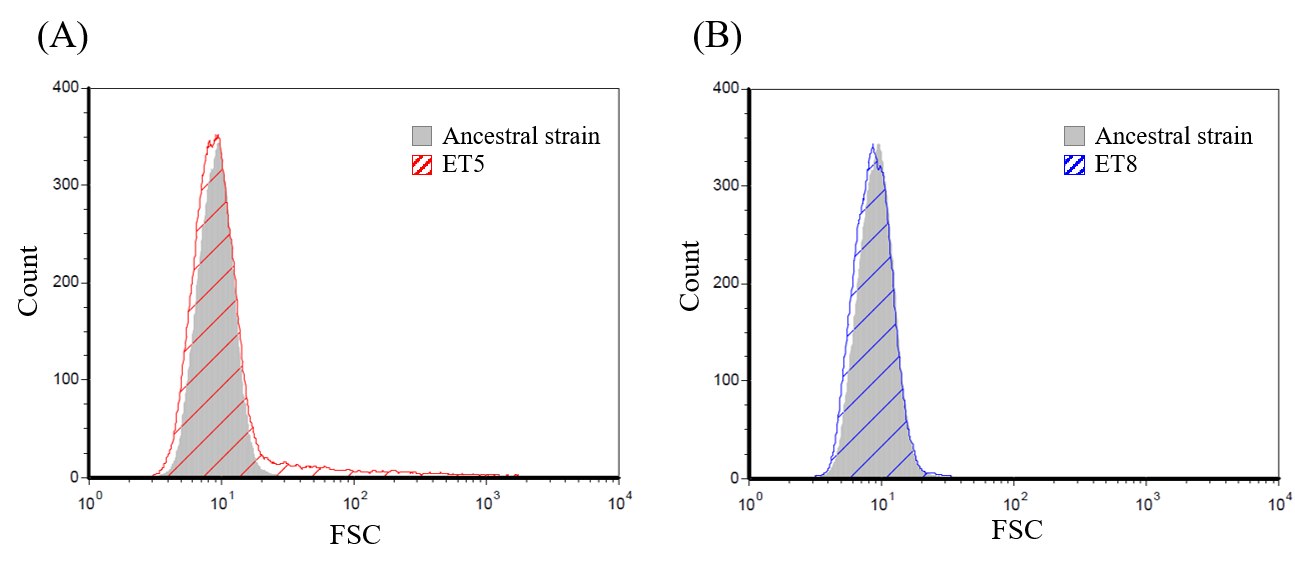


**Fig. S1.** Distribution of cell size in the batch-cultured ET5 strain compared with the ancestral strain by flow cytometry.

(Methods)

The ancestral strain and ET5 cells were grown in an M9-glucose medium with light illumination. Cells in log phase (4 h) were harvested by centrifugation (3,600 × g, 10 min) and washed twice with PBS solution (NaCl 8 g/L, KCl 0.2 g/L, Na_2_HPO_4_ 1.42 g/L, KH_2_PO_4_ 0.24 g/L, pH 7.4). The cells were mixed with 2.5 % glutaraldehyde for 2 h, then diluted in PBS solution at 1:1000 ratio. The cells were excited with laser (488 nm), then subjected to FACSCalibur^TM^ equipped with forward scatter detector (FSC) and side scatter detector (SSC) (BD Bioscience, Franklin Lakes, NJ 07417, USA) to analyze the cell size.

(Result)

The cell size distribution of the ET5 cell population was almost identical to that of the ancestral strain.

**Table S1**. List of all variations in the W3110 ancestral strain identified using NC_007779.1 as a reference.

| **Reference Position** | **Type** | **Length** | **Reference** | **Allele** | **Consequence** |
| --- | --- | --- | --- | --- | --- |
| 547694 | SNV | 1 | A | G |  |
| 547832 | Insertion | 1 | − | G |  |
| 556858 | SNV | 1 | A | T |  |
| 696892 | SNV | 1 | C | T |  |
| 987574 | SNV | 1 | G | T |  |
| 1093686 | SNV | 1 | T | C |  |
| 1197929 | SNV | 1 | C | A | *Icd* ^D401E^ |
| 1519222 | SNV | 1 | G | T |  |
| 1669599 | Deletion | 1 | C | − |  |
| 1979957 | SNV | 1 | G | T |  |
| 2005401 | SNV | 1 | C | T |  |
| 2245455 | SNV | 1 | A | G |  |
| 2945541 | SNV | 1 | G | A |  |
| 3268825 | SNV | 1 | A | G |  |
| 3276046 | SNV | 1 | G | T |  |
| 3746911 | SNV | 1 | G | A |  |
| 3755017 | SNV | 1 | T | A |  |
| 3958123 | Deletion | 32 | AN_30_T | − |  |
| 4371271 | Deletion | 2 | AA | − |  |
| 4591729 | SNV | 1 | C | T |  |

**Figure S2**


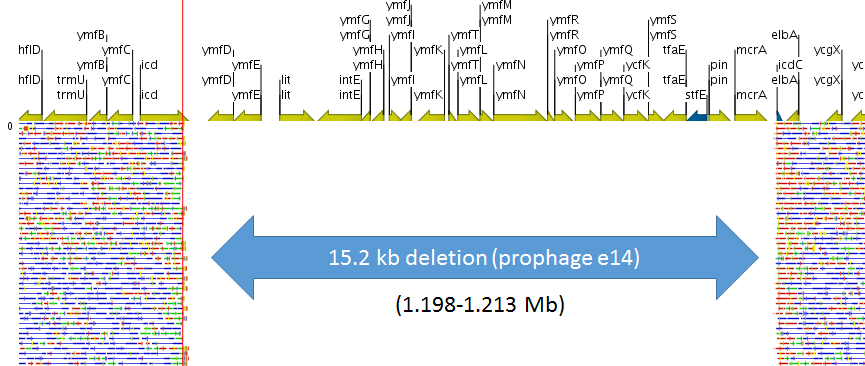


**Figure S2**. A 15.2-kb deletion identified as a zero-coverage region after mapping analysis. It might have been caused by a crossover between *icd* (Y75_p1106, 1251 bp) and *icdC* (pseudo; Y75_p1130, 153 bp). Sequence alignment showed that *icdC* is the C-term part of *icd* (93% nucleotide identities). The image was captured from the output screen of the CLC genomics workbench.
